# Letter to a Judge The implementation of the Law 8 / 2021 three years on

**DOI:** 10.1192/j.eurpsy.2025.1733

**Published:** 2025-08-26

**Authors:** M. Hernandez

**Affiliations:** MAPFRE, Madrid, Spain

## Abstract

**Introduction:**

Last scene of all That ends this strange eventful history, Is second childishness and mere oblivion, Sans teeth, sans eyes, sans taste, sans everything. Shakespeare, As you like it, II, vii, 163-166

**Objectives:**

The paper will make reference to the debate generated by the implementation of the Law 8/ 2021 approved three years ago in Spain. The objective was to provide support measures to persons with dementia according to their wishes, beliefs, values and preferences without resorting to legal incapacity. Respect to the person´s wishes is pivotal to the Law and is creating many problems due to the fact that some patients with severe cognitive impairment cannot make a judgement and is not clear what the judge can decide.

From the start, forensic psychiatrists received an increasing number of requests to produce medico-legal reports due to the omission to refer to the protective, medical and personal aspects that such measures involve. Some decisions totally ignore these aspects referring only to the patrimonial ones with no mention of the person`s life trajectory nor to the personal and medical care provided and the medication prescribed.

**Methods:**

In the second section, a case where the medical aspects where not contemplated in the sentence will illustrate the consequences for a patient who was misdiagnosed and mistreated.

**Results:**

An extensive observation following the Tavistock Method will describe the process, including the organizational dynamics.

**Conclusions:**

As Kitwood suggested, working with people suffering from dementia includes confronting organic impairment and the great difficulties there may be in consciously articulating any psychological conflicts, there may also be a breaking down of the individual’s lifelong defences that leaves the person exposed and vulnerable to episodes of catastrophic anxiety and rage. Fear of abandonment and inability to bear separateness are characteristic of dementia sufferers, and these persecutory states of mind increase with organic impairment. Caregivers, whether family or staff in residential or nursing homes, have a crucial function in containing those deteriorated aspects of the person they are with.

“Dementia will always have a deeply tragic aspect, both for those who are affected and for those who are close to them. There is, however, a vast difference between a tragedy in which persons are actively involved and morally committed, and blind and hopeless submission to fate” (Kitwood, 1997).

**Disclosure of Interest:**

None Declared

